# Heat-Killed *L. helveticus* Enhances Positive Mood States: A Randomized, Double-Blind, Placebo-Controlled Study

**DOI:** 10.3390/brainsci13060973

**Published:** 2023-06-20

**Authors:** Natsumi Mutoh, Izumi Kakiuchi, Kumiko Kato, Chendong Xu, Noriyuki Iwabuchi, Masayo Ayukawa, Kyoko Kiyosawa, Kazumi Igarashi, Miyuki Tanaka, Masahiko Nakamura, Mitsunaga Miyasaka

**Affiliations:** 1 Innovative Research Institute, Morinaga Milk Industry Co., Ltd., 1-83, 5-Chome, Higashihara, Zama-City 252-8583, Kanagawa, Japan; 2 Faculty of Nursing, Matsumoto College of Nursing, 3118, Sasaga, Matsumoto-City 399-0033, Nagano, Japan; 3 Department of Nursing, Matsumoto Junior College, 3118, Sasaga, Matsumoto-City 399-0033, Nagano, Japan; 4 Matsumoto City Hospital, 4417-180 Hata, Matsumoto-City 390-1401, Nagano, Japan

**Keywords:** gut-brain axis, psychobiotics, positive mood, paraprobiotics

## Abstract

When mood states are impaired, daily life is severely disrupted. To maintain a specific mood state, both positive and negative moods must be controlled; however, methods to maintain a positive mood have not been fully established. Previous studies have suggested that heat-killed *L. helveticus* MCC1848 has the potential to improve positive moods. This study aimed to test the efficacy of heat-killed *L. helveticus* MCC1848 in maintaining and improving a positive mood with PANAS, a questionnaire specifically designed to assess positive and negative mood, as the primary endpoint. Healthy Japanese nursing students (*n* = 46) were randomized to receive heat-killed *L. helveticus* MCC1848 (5 billion/day) or placebo powder for four weeks. Mood state was assessed before and two and four weeks after the intervention began; ingestion of heat-killed *L. helveticus* MCC1848 significantly improved PANAS ‘Positive Affect’ compared to the placebo. These results indicate that heat-killed *L. helveticus* MCC1848 is effective in enhancing positive mood.

## 1. Introduction

Impairment of mood state not only interferes with an individual’s daily life [1], but also causes significant losses to one’s career and education and ultimately to society through reduced work and study performance [2]. It has been reported that there are various types of “moods” [3]. In particular, negative and positive moods are not opposites, but independent dimensions with low correlation [4]. Importantly, both positive and negative moods need to be controlled in order to maintain specific mood states, but methods have not been fully established for improving positive mood compared to improving negative mood in foods and drugs [5].

The gut-brain axis is attracting attention as a point of action for mood control. The gut-brain axis refers to the concept in which the brain and gut, two distant organs, influence each other via the vagus nerve, circulatory system, and immune system [6,7,8]. It has also been shown that the gut microbiota influences the mood state of the host through the gut-brain axis [9]. “Psychobiotics” have been proposed as probiotics that provide mental health benefits through interaction with the gut microbiota [10] and are being studied worldwide. *Lactobacillus casei* strain Sirota (2.4 × 10^10^ cfu/day) has been reported to reduce anxiety symptoms in patients with chronic fatigue syndrome [11], and a combination of *Lactobacillus helveticus* R0052 and *Bifidobacterium longum* R0175 (3.0 × 10^9^ cfu/day) has been reported to reduce psychological distress [12]. Recently, researchers have proposed that the definition of ‘psychobiotics’ be extended to include exogenous effects on the brain via the gut microbiota [13]. This definition also includes inactivated microorganisms that affect mood states [14]. In fact, several effects of mood improvement with inactivated microorganisms have been reported. For example, heat-killed *L. gasseri* CP2305 (1.0 × 10^10^ cells/day) improved both State-Trait Anxiety Inventory (STAI) trait anxiety scores and salivary chromogranin A concentrations in a study of medical students preparing for national exams [15]. The majority of psychobiotics that have been reported thus far have been effective in suppressing negative moods, such as anxiety and depressive symptoms, as exemplified previously. On the other hand, a few bacteria have been shown to improve positive mood. In addition, inactivated microorganisms have several advantages over living organisms in several respects, such as (1) high storage stability, (2) no need for refrigeration, and (3) the ability to be incorporated into various food forms [16]. It is conceivable that materials with inactivated microorganisms that can improve positive mood inactivated could be useful in controlling daily mood states.

We previously reported that heat-killed *L. helveticus* MCC1848 may maintain a positive mood. When mood states were comprehensively assessed using the POMS 2 shortened version under conditions of temporary stress, the consumption of heat-killed *L. helveticus* MCC1848 improved the POMS 2 shortened version’s indicators of positive mood, namely, ‘Friendliness’ and ‘Vigor-Activity’ [17]. Heat-killed *L. helveticus* MCC1848 has been reported to suppress changes in gene expression in the nucleus accumbens, which animal experiments have shown to be activated during stress loading [18]. The nucleus accumbens plays an important role in positive emotions such as pleasure, reward, and motivation [19]. Recently, it was reported that increased dopamine levels in the nucleus accumbens play an important role in maintaining motivation to achieve difficult goals [20]. Impaired activity or damage to the nucleus accumbens is known to contribute to a lack of ‘preference’ or ‘pleasure’ [21] and anhedonia [22]. Indeed, an animal study showed that heat-killed *L. helveticus* MCC1848 improved anhedonia of pleasure, but not polydipsia, another endophenotype of depression [18]. These studies suggest that heat-killed *L. helveticus* MCC1848 acts on the nucleus accumbens and improves positive mood. On the other hand, the POMS 2 shortened version is a questionnaire suitable for comprehensive assessment of a wide range of mood states and was used as an assessment measure in previous clinical trials [23]. However, to specifically assess any ‘positive mood’-improving effects of heat-killed *L. helveticus* MCC1848, it is necessary to conduct a confirmatory study using a questionnaire that is better suited to assessing positive mood.

In this study, a confirmatory clinical trial was conducted using the PANAS questionnaire as the primary endpoint; the PANAS is one of the most widely used scales for assessing positive and negative affect, and the reliability and validity of the Japanese version have been demonstrated [24]. The results of this study suggest that heat-killed *L. helveticus* MCC1848 specifically improves positive moods in healthy adults.

## 2. Materials and Methods

### 2.1. Trial Design

This study was a randomized, double-blind, placebo-controlled, parallel-group clinical trial conducted at Matsumoto Junior College (Matsumoto City, Nagano, Japan) from March to April 2022 to investigate the effects of heat-killed *L. helveticus* MCC1848 intake on mood. For students preparing for clinical training, this is the most stressful time of the year. The participants received daily pre-clinical training between April and May 2022. Participants in this study had a 1-week preintervention period and a 4-week intervention period. During the 1-week preintervention period, the participants were instructed to keep a daily diary. The participants were then randomly assigned to one of two groups: the placebo or *L. helveticus* group. During the intervention period, the participants consumed the specified product once daily for 4 consecutive weeks. The participants were surveyed using a questionnaire in the preintervention period and 2 and 4 weeks after the start of the intervention. The participants were asked not to change their usual eating habits and health-related behaviors during the study period. During the study period, the participants were asked to record their daily intake of the test food and the occurrence of any unusual events, such as medication use or adverse events, to ensure compliance with the study. The study protocol was conducted in accordance with the Helsinki Declaration of 1975 as revised in 2013 and the Ethical Guidelines for Medical and Health Research Involving Human Participants proposed by the Ministry of Education, Culture, Sports, Science, and Technology and the Ministry of Health, Labor, and Welfare, and received the approval of the Ethical Advisory Committee of Matsumoto Junior College. This study was registered in the UMIN Clinical Trial Registry before commencement (UMIN000047065).

### 2.2. Participants

Participants were recruited from a cohort of healthy third-year nursing students at Matsumoto Junior College (Matsumoto City, Nagano, Japan). The inclusion criterion was an age between 20 and 64 years old. Participants were excluded if they (1) regularly used medicines, supplements, and other foods containing added lactic acid bacteria and Bifidobacterium (excluding foods in which these bacteria naturally occur such as yogurt); (2) regularly took medicines or supplements that affect mood and fatigue; (3) participated in other drug or food clinical trials within the previous month; (4) had a history of serious disorders of the liver, kidney, heart, lung, gastrointestinal, blood, endocrine, or metabolic systems; (5) had a history of drug or severe food allergies; (6) were pregnant, lactating, or expected to be pregnant during the study; or (7) were deemed inappropriate for participation in this trial by the investigator based on participant background, physical examination, etc.

Prior to the initiation of this study, the procedure was fully explained to the participants, and each participant provided written informed consent to participate. Prior to commencing the study, participants were required to complete a comprehensive health assessment questionnaire and to not take any regular medication. The participants were enrolled after satisfying all inclusion and exclusion criteria for eligibility to participate in the present study by questionnaire and interview. The participants were blinded to the treatment allocation.

### 2.3. Intervention

The participants were given either placebo or heat-killed *L. helveticus* MCC1848 (strain happiness) powder. Heat-killed *L. helveticus* MCC1848 powder was formed into a stick containing 5 × 10^9^ heat-killed *L. helveticus* MCC1848 cells, the same dose as in the study conducted by Mutoh et al. [17], with maltodextrin as the excipient, and was taken once daily. The placebo powder contained maltodextrin. The test foods could not be distinguished by their packaging, taste, color, or odor, and the treatment allocation condition was hidden from the participants and study staff. The test foods were supplied by Morinaga Milk Industry Co., Ltd. (Tokyo, Japan).

### 2.4. Outcomes

The scores for all efficacy assessments were compared at two points: before the intervention and during the intervention. Scores during the intervention were the average of the scores at 2 and 4 weeks after the intervention began. The PANAS [24] questionnaire score during the intervention period was used as the primary outcome. The secondary outcomes measured were the POMS 2 [23], visual analog scale (VAS) for ‘feeling tired’, ‘being exhausted’, ‘feeling good’ and ‘being relaxed’, acute form of the SF-36v2 [25], Chalder Fatigue Scale (CFS) [26], and Athens Insomnia Scale (AIS) [27] scores during the intervention period.

Safety assessments were carried out for all participants who consumed the study food on at least one occasion. During the study period, all adverse events related to the participant and objective symptoms were recorded in the participant’s diary.

### 2.5. Sample Size

On the basis of previously published studies of heat-killed lactic acid bacteria showing positive effects on mood, fatigue, and sleep in healthy participants [15,28,29], it was estimated that the inclusion of approximately 30 participants per treatment group would be necessary to draw firm conclusions. An earlier clinical study (in press) of heat-killed *L. helveticus* MCC1848 was also conducted with a sample size of 30 participants per group. This sample size (30) was used in the present study.

### 2.6. Randomization

An independent allocation manager with no other involvement in the conduct of the study randomly allocated eligible participants to the placebo or *L. helveticus* groups by means of a computer-generated randomization script that stratified participants by both their fatigue-inertia (FI) score on the shortened version of POMS 2 (greater than or equal to 50; less than 50) and AIS score (greater than or equal to 4; less than 4). These factors have been reported to have a significant impact on mood [30].

Both the participants and observers were blinded with regard to the group allocation. Double-blinding was achieved by labeling the test food boxes with a specific identification number only. The blinding code was held by the allocation manager and opened only after the completion of the study.

### 2.7. Statistical Methods

The primary population was the per protocol set (PPS) population, defined as all randomly assigned participants who consumed the test food for 60% of the test period.

All data are shown as the means ± standard deviations. All statistical analyses were performed using IBM SPSS Statistics (ver. 24.0). *p* values of less than 0.05 were considered to indicate statistical significance. Differences in background characteristics at screening, namely, the PANAS, POMS 2, VAS, SF-36v2, CFS, and AIS scores, were evaluated with Student’s *t*-tests between groups. Paired *t*-tests were used to identify differences within groups at different time points. In the correlation analysis, Pearson’s product moment correlation coefficient was calculated.

For the safety assessment, all participants who consumed the test food were included in the analysis population. For each group, the number of participants, the number of adverse events, and their incidence rate (i.e., frequency calculated as the number of confirmed adverse events/total number of participants) were calculated. Differences in incidence rates between the groups were tested with Fisher’s exact test, and *p* values were calculated.

The time tolerance with regard to the data was set at ±1 week. Missing data were treated as missing values.

## 3. Results

### 3.1. Participant Flow

**Figure 1:**
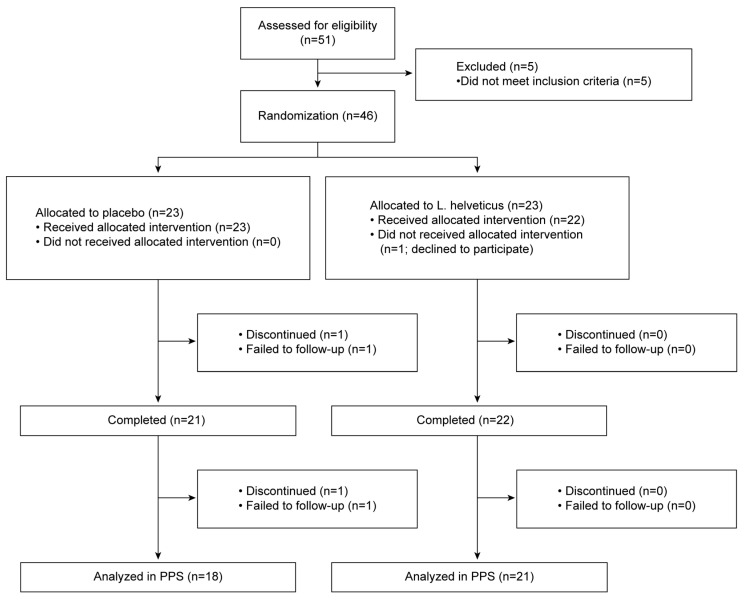
Flowchart of participant inclusion. PPS: per protocol set.

The participant flow diagram is shown in Figure 1. Fifty-one participants expressed an interest in participating in this study, and 46 were considered eligible. A total of 46 participants were randomized into two groups. Of these participants, one declined to participate and withdrew consent prior to the consumption of the test food. One was lost to follow-up and 43 completed the study. In the PPS analysis, four participants were excluded because they did not meet the inclusion criteria or had low compliance, i.e., <60% test food intake during the test period. Therefore, data from 39 participants in the PPS were included in the efficacy analysis.

### 3.2. Baseline Data and Compliance

Table 1 shows the baseline participant characteristics. There were no significant differences in any of the baseline characteristics between the two groups.

The mean self-reported compliance rates were 97.4% in the placebo group and 96.0% in the *L. helveticus* group; however, the difference was not statistically significant.

### 3.3. Effects of L. helveticus MCC1848 on PANAS Scores

The PANAS results are shown in Table 2. Increased scores for ‘Positive Affect’ and ‘Negative Affect’ indicate increased positive and negative mood, respectively. As we had predicted, ‘Negative Affect’ scores remained unchanged during the intervention, whereas ‘Positive Affect’ scores were significantly higher in the *L. helveticus* group than in the placebo group. These results suggest that heat-killed *L. helveticus* MCC1848 can specifically improve positive mood without affecting negative mood.

### 3.4. Effects of L. helveticus MCC1848 on POMS 2, VAS, Sleep, Fatigue and Other QOL Scores

The secondary endpoint scores are summarized in Table 3. In the *L. helveticus* group, the POMS 2 ‘Confusion-Bewilderment’ score improved significantly during the intervention compared to that before the intervention. No significant differences were found between the two groups in any of the secondary endpoints.

### 3.5. Safety

During the study, a total of 19 adverse events were reported (eight events in the placebo group; 11 in the *L. helveticus* group). The most common adverse event was abdominal pain (nine events). All the adverse events were mild and transient and were not associated with consumption of the test foods, as confirmed by the physician following the health survey during the study period. The incidence of adverse events did not differ significantly between the two groups (placebo: 26.1%; *L. helveticus*: 21.7%).

## 4. Discussion

Heat-killed *L. helveticus* MCC1848 was previously shown to improve the scores on positive mood items in the POMS 2 shortened version in a previous clinical study (Mutoh et al. [17], in press); therefore, a confirmatory study was conducted to determine whether heat-killed *L. helveticus* MCC1848 truly improves positive mood status. In the current study, the significant improvement in the POMS 2 shortened version seen in the previous study was not observed, but there was a significant improvement in PANAS ‘Positive Affect’, which correlated with the positive mood item scores of the POMS 2 shortened version (vs. POMS2 ‘Vigor-Activity’ r = 0.798, *p* < 0.001, vs. ‘Friendliness’ r = 0.482, *p* < 0.001, Figure S1). For reference, details of the PANAS “Positive Affect” scores are provided in Table S1. No significant differences were found in PANAS ‘Negative Affect’ scores. Heat-killed *L. helveticus* MCC1848 was found to specifically improve positive, but not negative, affect.

We consider that the design of the current study is appropriate for evaluating the effect of heat-killed *L. helveticus* MCC1848 on transient stress. As an evaluation system with a transient stress load, prepractice students were included. This is the same participant cohort that has been used in previous studies to confirm the effects of heat-killed *L. helveticus* MCC1848 (Mutoh et al. [17], in press). This cohort is also commonly used in studies on the effects of other bacteria [29,31,32,33], and we consider it an appropriate transient stress load model. With the aim of assessing positive and negative moods, the primary endpoint in this study was the PANAS, which is one of the most widely used scales for assessing positive and negative affect [34]. The Japanese version of the PANAS was developed and evaluated by Sato and Yasudu [24] in 2001, and its reliability and validity have been demonstrated. POMS 2, which was the primary endpoint of the previous study (Mutoh et al. [17], in press), is a questionnaire that can broadly assess mood states according to eight different scales. In comparison, the PANAS is a questionnaire with only two scales: positive affect (PA) and negative affect (NA). Using the PANAS, we were able to conduct a confirmatory study to assess the positive and negative mood effects of heat-killed *L. helveticus* MCC1848. The design of the study was appropriate to assess the effects of heat-killed *L. helveticus* MCC1848 on transient stress.

In a previous study, the POMS 2 shortened version ‘Friendliness’ measure showed a significant improvement, and the ‘Vigor-Activity’ measure showed a trend toward improvement (Mutoh et al. [17], in press), but no significant changes were observed in either of these endpoints in the current study. For ‘Vigor-Activity’, the difference in scores between the placebo and *L. helveticus* groups in the previous study was 4.0, while the score difference in the current study was 3.1. Because the difference in score differences is not substantial, we believe that there was a certain effect of heat-killed *L. helveticus* MCC1848 on ‘Vigor-Activity’. On the other hand, for ‘Friendliness’, the difference in scores between the placebo and *L. helveticus* groups in the previous study was 4.7, while the same score difference in the current study was −0.6. It was expected that ‘Friendliness’ scores would be influenced by lifestyle, particularly in considering the nature of the questions (e.g., ‘I enjoy socializing’, ‘I care about others’ etc.) assessing ‘Friendliness’. Therefore, a correlation analysis of the ‘Friendliness’ score with the lifestyle responses given at baseline was conducted to determine the factors influencing the ‘Friendliness’ score. The ‘Friendliness’ score tended to correlate positively with the ‘having part-time jobs’ status (Table S2), suggesting that having part-time jobs is associated with an increase in the ‘Friendliness’ score. In the present study, the proportion of participants with part-time jobs was significantly higher than that in the previous study (Table S3), which may have prevented the effects of the food intervention. The effect of heat-killed *L. helveticus* MCC1848 on POMS 2 ‘Friendliness’ could be determined from the present study. Whether there is an effect of heat-killed *L. helveticus* MCC1848 on POMS 2 ‘Vigor-Activity’ and ‘Friendliness’ should be investigated further by conducting future clinical studies.

There are several reports of reduced work performance due to mood disorders, with 65.5 days of work loss per year for bipolar disorder and 27.2 days per year for major depressive disorder [2]. It is considered necessary to control mood states on a daily basis to maintain productivity. Several probiotics and bacterial bodies of microorganisms have been reported to affect mood and brain function, but most of them have the effect of suppressing negative moods [11,12], and few have been reported to improve positive moods. Negative and positive moods are not opposites, but rather independent dimensions with low correlations; decreasing negative mood does not mean that positive mood will increase accordingly [4]. Indeed, analysis of the data from the current study did not show a correlation between the PANAS ‘Positive Affect’ and ‘Negative Affect’ measures (r = 0.151, *p* = 0.187, Figure S1). To maintain a mood state, both positive and negative moods must be properly controlled. The findings of this study, namely, that heat-killed *L. helveticus* MCC1848 improved positive mood, are of great significance to society.

This study had several limitations. The study had a skewed male-to-female ratio of 4:35. The number of participants in this study was smaller than the sample size at the time of planning. In addition, the study was conducted using a questionnaire, and there were no objective assessment items. We believe that adding a test with objective indices (e.g., cortisol in saliva) would help to elucidate the mechanism of action in humans and enhance the certainty of the results of this study.

## 5. Conclusions

The results of this study suggest that heat-killed *L. helveticus* MCC1848 maintains a positive mood in healthy adults. Because of the bias resulting from the predominance of young women among the subjects, additional studies should be conducted with a wider range of subjects.

## Figures and Tables

**Table 1:** Baseline characteristics of subjects.

Variables	Placebo (*n* = 18)	*L. helveticus* (*n* = 21)
Age (years) ^1^	21.9 ± 4.4	21.6 ± 5.9
Sex (male/female) ^2^	2/16	2/19
Smoking habit (*n*, %) ^2^	0 (0)	0 (0)
One-person households (*n*, %) ^2^	2 (11.1)	6 (28.6)
Having part-time jobs (*n*, %) ^2^	8 (44.4)	13 (61.9)
Habitual yogurt intake (*n*, %) ^2^	10 (55.6)	11 (52.4)

^1^ Values are means ± standard deviation. Scores of each group were compared by Student’s *t*-test. No significant differences were found, all *p* > 0.05. ^2^ Scores of each group were compared by Fisher’s exact test. No significant differences were found, all *p* > 0.05.

**Table 2:** The Positive and Negative Affect Schedule (PANAS) scores before and during the intervention ^1,2^.

	Before Intervention			During Intervention		
	Placebo	*L. helveticus*	*p* Value (Inter-Group)	Placebo	*L. helveticus*	*p* Value (Inter-Group)
Positive Affect	23.2 ± 5.9	25.5 ± 6.5	0.258	22.1 ± 5.9	26.7 ± 7.1	0.034
Negative Affect	21.5 ± 5.4	20.7 ± 7.6	0.715	19.9 ± 5.1	19.8 ± 7.5	0.968

^1^ Values are means ± standard deviation. ^2^ Inter-group differences were compared by Student’s *t*-test. Intra-group differences were compared using the paired *t*-test. No significant intra-group differences were found, all *p* > 0.05.

**Table 3:** Secondary endpoints before and during the intervention ^1,2^.

Variables	Before Intervention			During Intervention		
	Placebo	*L. helveticus*	*p* Value (Inter-Group)	Placebo	*L. helveticus*	*p* Value (Inter-Group)
POMS2						
Total mood disturbance	48.9 ± 7.8	46.2 ± 9.7	0.347	46.6 ± 6.2	44.2 ± 8.5	0.319
Anger-hostility	45.1 ± 5.9	43.6 ± 7.7	0.507	43.8 ± 5.7	42.0 ± 5.3	0.308
Confusion-bewilderment	50.8 ± 7.5	50.5 ± 8.9	0.894	48.8 ± 7.9	47.8 ± 8.1 ^§^	0.708
Depression-dejection	50.3 ± 7.4	47.2 ± 7.9	0.217	47.5 ± 5.9	46.1 ± 6.9	0.502
Fatigue-inertia	47.7 ± 9.7	46.0 ± 9.9	0.610	45.7 ± 6.7	44.7 ± 8.9	0.718
Tension-anxiety	51.3 ± 9.1	48.3 ± 10.0	0.316	50.5 ± 8.8	46.9 ± 9.4	0.227
Vigour-activity	50.2 ± 7.9	51.8 ± 10.0	0.577	50.6 ± 5.5	53.7 ± 9.6	0.235
Friendliness	56.1 ± 5.9	56.0 ± 9.6	0.952	55.9 ± 6.0	55.3 ± 7.8	0.788
SF-36v2						
Physical component summary	57.1 ± 9.8	55.4 ± 6.8	0.543	57.1 ± 6.0	56.8 ± 4.5	0.830
Mental component summary	50.0 ± 8.2	53.3 ± 11.0	0.302	48.3 ± 6.3	52.8 ± 10.6	0.120
Role component summary	49.1 ± 10.5	49.5 ± 7.1	0.897	50.9 ± 9.2	49.1 ± 8.0	0.507
VAS						
“Feeling tired”	44.0 ± 27.0	38.5 ± 22.4	0.493	45.6 ± 23.4	43.1 ± 23.7	0.744
“Being exhausted”	37.9 ± 26.4	28.6 ± 27.6	0.292	37.3 ± 24.0	34.8 ± 26.1	0.755
“Feeling good”	60.9 ± 23.1	64.3 ± 21.5	0.638	62.3 ± 18.8	64.1 ± 23.4	0.790
“Being relaxed”	63.3 ± 18.8	60.0 ± 25.3	0.648	61.0 ± 18.5	61.9 ± 21.8	0.890
Athens insomnia scale	4.3 ± 3.0	4.0 ± 2.9	0.730	4.3 ± 2.2	4.1 ± 4.0	0.826
Chalder fatigue scale	14.9 ± 6.2	13.0 ± 7.4	0.370	13.7 ± 6.9	12.5 ± 7.7	0.632

^1^ Values are means ± standard deviation. ^2^ Inter-group differences were compared using Student’s *t*-test. ^§^ *p* < 0.05 when compared to before intervention. No significant inter-group differences were found, all *p* > 0.05. Intra-group differences were compared based on *p* values.

## Data Availability

The data presented in this study can be found in this published article and its Supplementary Information Files.

## Supplementary Materials

The following supporting information can be downloaded at: https://www.mdpi.com/article/10.3390/brainsci13060973/s1, Table S1. Data for each question item in the positive mood indicator ^1,2^; Table S1: Data for each question item in the positive mood indicator.; Table S2: Correlation analysis of the ‘Friendliness’ score with lifestyle responses.; Table S3: Comparison of percentages of subjects with part-time jobs.; Figure S1: Correlation between PANAS and POMS2 items.
