# Supplementary material for: Heat-Killed L. helveticus Enhances Positive Mood States: A Randomized, Double-Blind, Placebo-Controlled Study
Source: Brain Sci. 2023 Jun 20;13(6):973. doi: 10.3390/brainsci13060973 (PMC10296070; doi:10.3390/brainsci13060973)
Supplement: Supplementary file 1 [file brainsci-13-00973-s001.zip › brainsci-2445328-supplementary.pdf]

Supplementary Materials

Table S1. Data for each question item in the positive mood indicator.[1,2]

PANAS 'Positive affect'	Before intervention		During intervention	
	Placebo	L. helveticus	Placebo	L. helveticus
Active	3.5 ± 1.1	3.7 ± 1.1	3.2 ± 0.8	3.9 ± 1.0*
Proud	2.3 ± 0.9	3.1 ± 1.1*	2.5 ± 0.8	3.2 ± 1.2
Strong	2.9 ± 1.1	3.0 ± 1.3	2.6 ± 0.8	3.0 ± 1.2
Determined	3.2 ± 1.1	3.2 ± 1.2	2.9 ± 0.9	3.2 ± 1.2
Inspired	2.8 ± 1.2	3.1 ± 1.2	2.9 ± 0.8	3.5 ± 1.2
Excited	3.4 ± 1.4	4.1 ± 1.1	3.0 ± 1.1	4.0 ± 1.1*
Alert	2.8 ± 0.9	2.9 ± 1.2	2.8 ± 0.6	3.0 ± 0.9
Enthusiastic	2.2 ± 1.0	2.4 ± 1.0	2.4 ± 1.1	2.6 ± 0.9

[1] Values are means ± standard deviation of the mean.

[2] Scores of each group before and during intervention were compared by Student's t-test.

* $P < 0.05$ between the two groups.

Table S2. Correlation analysis of the 'Friendliness' score with lifestyle responses.[1,2]

	Pearson's coefficient of correlation	*p* value
Age	0.114	0.268
Sex	-0.067	0.515
One-person households	-0.086	0.404
Having part-time jobs	0.225	0.027
Habitual yogurt intake	0.065	0.526

[1] Baseline scores from the current and previous studies [17] were used.

[2] Pearson's correlation analysis was performed. Smoking was excluded from the analysis because it was not suitable for inclusion due to the small number (total of 3 cases) of occurrences.

Table S3. Comparison of percentages of subjects with part-time jobs.[1]

	Current study	Previous study [17]	*p* value
Having part-time jobs (n, %)	21 (53.8 %)	19 (32.8 %)	0.039

[1] Chi-square test performed.

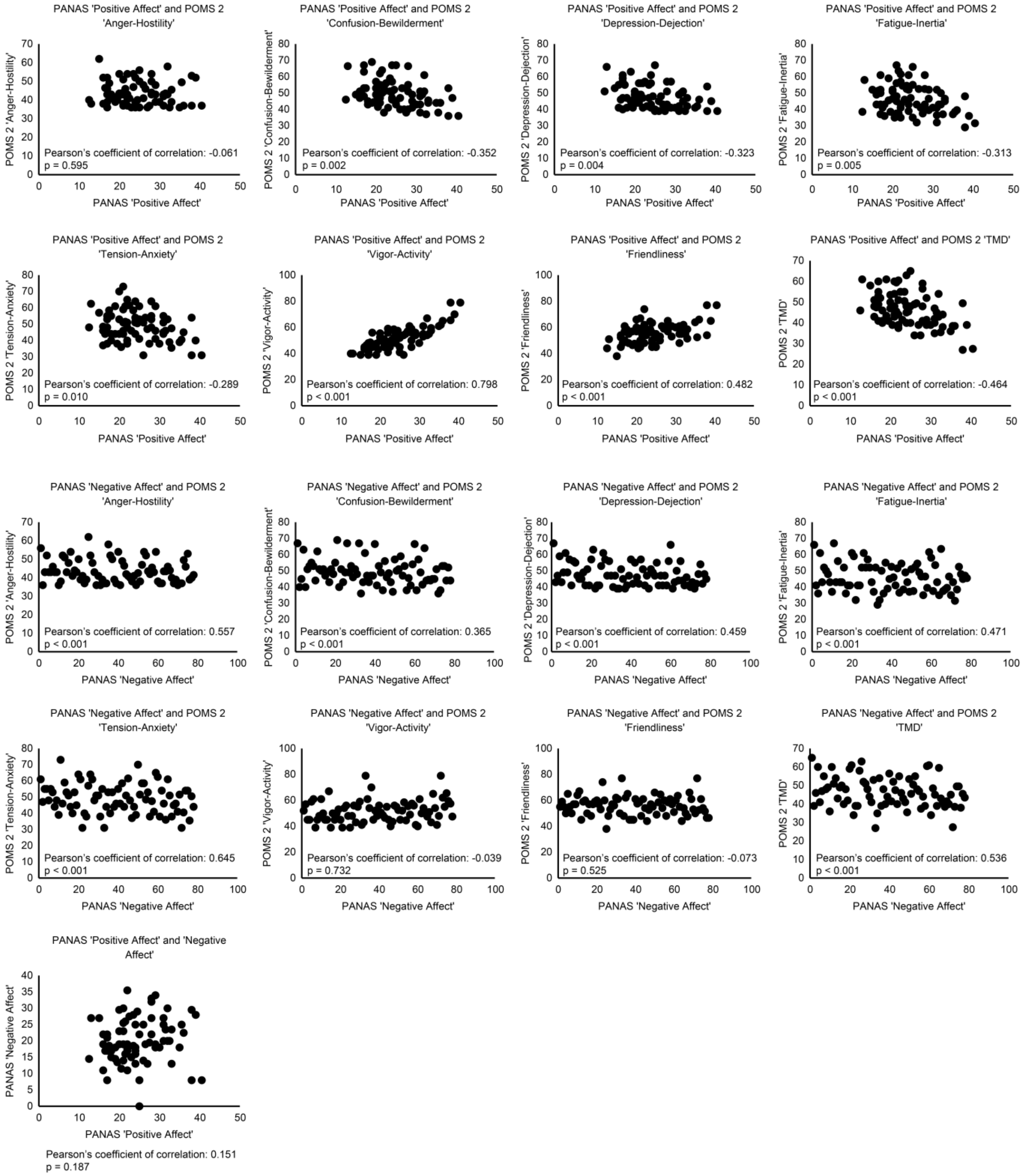

Figure S1. Correlation between PANAS and POMS2 items.

Pearson's correlation coefficients and significance probabilities were calculated for each of the PANAS and POMS 2 items.
